# Metabarcoding insights into the diet and trophic diversity of six declining farmland birds

**DOI:** 10.1038/s41598-021-00519-9

**Published:** 2021-10-26

**Authors:** Xabier Cabodevilla, François Mougeot, Gerard Bota, Santi Mañosa, Francesc Cuscó, Julen Martínez-García, Beatriz Arroyo, María J. Madeira

**Affiliations:** 1 Department of Zoology and Animal Cell Biology, Faculty of Pharmacy, University of the Basque Country (UPV/EHU), Paseo de la Universidad 7, 01006 Vitoria-Gasteiz, Alava Spain; 2 Instituto de Investigación en Recursos Cinegéticos (IREC) (CSIC-UCLM-JCCM), Ronda de Toledo 12, 13005 Ciudad Real, Spain; 3 Landscape Dynamics and Biodiversity Programme, Forest Science and Technology Center of Catalonia (CTFC), Solsona, Catalonia Spain; 4 Departament de Biologia Evolutiva, Ecologia I Ciències Ambientals, Institut de Recerca de la Biodiversitat (IRBio), Universitat de Barcelona, Avinguda Diagonal 643, 08028 Barcelona, Catalonia Spain

**Keywords:** Conservation biology, Zoology

## Abstract

Knowledge of feeding ecology of declining species, such as farmland birds, is essential to address their conservation requirements, especially when their habitats are suffering important reductions of trophic resources. In this study, we apply a metabarcoding approach to describe the diet composition of six of the most significant farmland birds inhabiting European cereal pseudo-steppes: little bustard, great bustard, pin-tailed sandgrouse, black-bellied sandgrouse, red-legged partridge, and common quail. We further studied seasonal diet variations (autumn to spring) in all species but the common quail, whose diet was studied during spring and summer. We show that study species´ diets mostly consisted of plants, although in the case of little bustard and great bustard arthropods are also highly relevant. Among arthropods, we found high proportions of thrips, arachnids, and springtails, which were previously unreported in their diet, and some taxa that could be used as antiparasitic food. Moreover, we report that little bustard’s diet is the least rich of that of all studied species, and that diet of all these species is less diverse in winter than in autumn and spring. Diet composition of these declining species supports the importance of natural and semi-natural vegetation and landscape mosaics that can provide a wide variety of arthropods, plants, and seeds all year-round.

## Introduction

In recent decades, agricultural landscapes have changed dramatically worldwide^1^. Since the 1960s, farmland intensification supported by subsidies of the European Common Agricultural Policy (CAP), which involved mechanization and wide use of synthetic fertilizers and phytosanitary products, has changed agricultural landscapes in Europe. After the 1990s, agricultural intensification was further increased by the globalization of commodity markets and CAP reforms, leading in turn to land abandonment in less productive areas^2^.

All these processes led, under most circumstances, to a homogenization of agricultural landscapes by creating larger plots with sparse natural or semi-natural elements^3^. Unfortunately, agricultural intensification has had severe consequences for biodiversity^4–6^ and it is considered the main driver of the current biodiversity loss in Europe^4^. Farmland birds are an excellent example of this, as their populations have strongly declined across Europe over the last decades during the process of agriculture intensification^7^, despite multiple reforms of EU’s CAP to try to reduce the environmental impacts of modern farming^8^. Agricultural intensification has led to a reduction of fallow lands (agricultural plots that are not cultivated during one or more growing seasons), which are important habitats for farmland birds^8–10^. This has been very relevant in the Iberian Peninsula, which is a European hotspot for steppe birds owing to its biogeography (Mediterranean region), arid environment and agriculture landscape (characterized by traditional rain-fed winter cereal‐based cultivation, crop rotations, low intensification, and relatively high proportion of fallows) ^8,9,11,12^. In the Iberian Peninsula, where the soil is poor and artificial fertilizers seldom used until recently, a crop rotation system including fallows was widespread practice ^13^. However, fallow land in Spain has decreased by 1.1 million hectares in the last 15 years^8,13^, leading to more homogeneous landscapes, with strong negative consequences for steppe birds and farmland biodiversity. These changes have had negative impacts on the availability of trophic resources for steppe birds, with reductions in plant and insect abundance and species richness^5,14–16^ and consequently seed banks^14,17^ and seed availability^18^.

Considering that more than 80% of steppe bird species have an unfavorable conservation status (i.e. worse than “least concern”) at the European level^19^ and that agricultural intensification has a direct negative impact on the ecosystem's trophic resources and their availability^5,7,14,15,17^, knowledge of the trophic ecology of these species takes on special relevance. The study of feeding ecology is essential to understand the biological and ecological requirements of species and, therefore, proper management and conservation of their populations and habitats. Unfortunately, the information available on the trophic ecology of many declining steppe birds is limited^20,21^. The few studies that exist describe the diet of some of these species based on visual (micro- and/or macroscopic) studies of crops, stomachs, or feces contents^22–24^. These techniques may have a strong bias since the detection probability is strongly influenced by digestion, even with the impossibility of detecting some taxa in feces due to their complete digestion^25^. Unlike the traditional techniques for diet study, the modern genetic methods through fecal eDNA metabarcoding can easily go beyond the detection capabilities of visual identification^25,26^. These methods are becoming increasingly popular for the study of diet and, although they also have some important biases regarding amplification process^25,26^, they allow a highly accurate description of species trophic ecology^25^.

This study aims to describe the diet of some of the most significant steppe bird and farmland game bird species of the European cereal pseudo-steppes through DNA-metabarcoding of species´ fecal samples. All these farmland birds inhabit increasingly intensified agricultural landscapes and are currently declining^8,27^ so there is an urgent need to improve knowledge about their diet to correctly understand their trophic requirements and conservation needs. We assessed the diet of four steppe birds, the little bustard (*Tetrax tetrax*), the great bustard (*Otis tarda*), the pin-tailed sandgrouse (*Pterocles alchata),* and the black-bellied sandgrouse (*Pterocles orientalis*) and two farmland game birds, the red-legged partridge (*Alectoris rufa*) and the common quail (*Coturnix coturnix*). The diet of all these species is not yet well known, and here we focused on the relative importance of plants and invertebrates (Annelida, Arthropoda, and Mollusca) in their diet. With this level of taxonomic resolution, we compared diet composition and richness between species. For four species that could be sampled from autumn to spring, we also describe seasonal variations in diet. We provide the most up-to-date comprehensive understanding of their food requirements in a rapidly changing environment.

## Results

We achieved a mean coverage of 130,006 reads (min: 41,632, max: 227,268). On average, diet reads accounted for 69.58% of the total reads. Diet reads were classified into 879 unique OTUs, 636 OTUs of plants, 235 OTUs of arthropods, 3 OTUs of annelids, and 5 OTUs of mollusks. The remaining reads (not accounted as diet) mostly belonged to DNA from the host species itself, parasites or fungi (probably environmental contamination) and low-quality sequences that could not be accurately identified.

**Figure 1:**
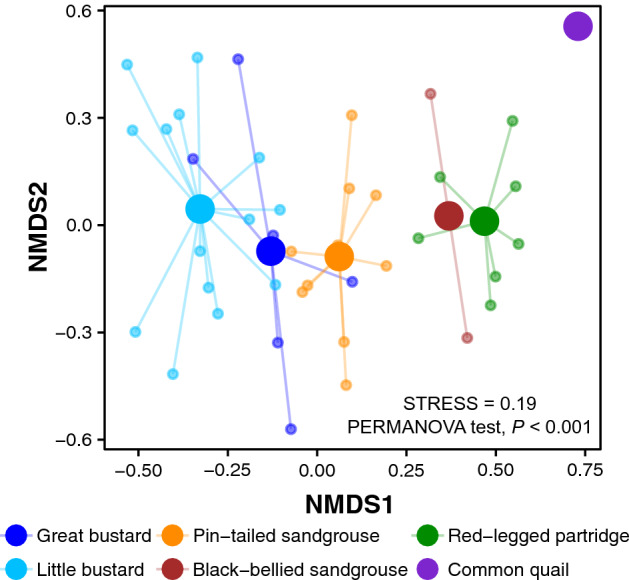
Graphical illustration of the results of the NMDS analysis of the studied birds’ diet (OTUs). Larger dots represent the centroid of the flocks of each species, while smaller dots represent the individual flock data of each species.

We found clear differences in diet composition between studied species based on the results of the Non-metric multidimensional scaling (NMDS) analysis (Fig. 1 NMDS axis 1) and PERMANOVA test (*F*_5,39_ = 2.64, *P* < 0.001; Fig. 1). Similar results were obtained considering just presence data (Supplementary Fig. S1 online). The diet of both bustard species was more similar to each other than to those of sandgrouse and partridges. In the case of common quail, although we only had one sample, its breeding season diet appeared to be quite different from the non-breeding diet of the other species according to both NMDS axis (Fig. 1).

**Figure 2:**
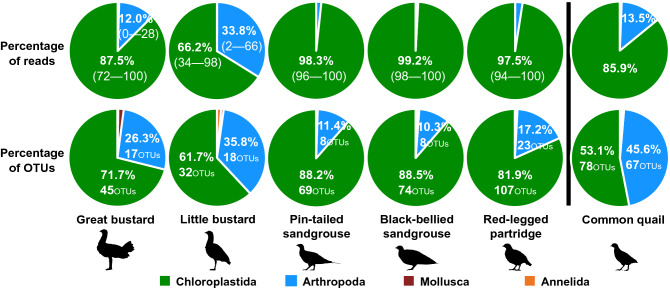
Mean percentage of reads (a measure of abundance) and OTUs (a measure of richness) of the Chloroplastida, Arthropoda, Mollusca, and Annelida taxa present on the diet of studied farmland bird species. Percentages > 10% are specified numerically and in the case of reads, SD is indicated in brackets. In the OTUs graphs, OTUs richness is also indicated. Note that samples of great bustard, little bustard, pin-tailed sandgrouse, black-bellied sandgrouse, and red-legged partridges were collected during the non-breeding season, whereas samples of common quail were collected during the breeding season.

**Figure 3:**
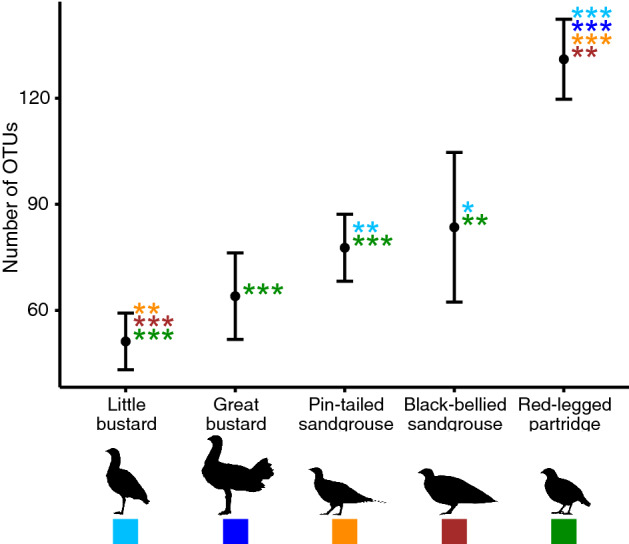
OTUs richness in the diet of studied species. The whiskers represent the 95% confidence intervals. The statistical significance of the Tukey post-hoc test is provided by * symbols. ***P < 0.001. **P < 0.01. *P < 0.05. The colour of the * corresponds to the colour of the species with which the comparison is made. For those comparisons that were not statistically significant, no significance data are provided. For detailed statistical results of Tukey post-hoc tests, see Supplementary Table S2 online.

Differences between species were also clear when we checked the proportion of reads and OTUs of each taxon obtained from sequencing (Figs. 2 and 3). The results showed that the plant material was the most important component of the diet of all six species, but the importance of invertebrates in the diet strongly differed among them (Fig. 2). Invertebrates contributed to the diet of all species, but they were especially important for both bustard species in autumn, winter, and spring and common quail during the breeding season. In all species, the contribution of invertebrates to diet was mostly made up by arthropods, while mollusks and annelids seemed to be rarely taken.

The ANOVA test showed that there were significant differences in OTU richness among species (*F*_4,34_ = 35.45, *P* < 0.001; Fig. 3). During the non-breeding season, the diet of red-legged partridges was the richest, with an average of 131 OTUs (95% Confidence Interval (CI) = 120–142 CI), followed by the diets of black-bellied sandgrouse (84 OTUs, 62–1 05 CI), pin-tailed sandgrouse (78 OTUs, 68–87 CI) and great bustard (64 OTUs, 52–76 CI). The diet of little bustard (51 OTUs, 43–59 CI) was the least rich. In the case of the two bustard species, around 30% of the OTUs belonged to Arthropoda taxa (Fig. 2). In contrast to the large differences found among species, we found low variability in OTU richness within species, as shown by the 95% Confidence Intervals (Fig. 3), even if samples were collected in different seasons or different geographical regions. During the breeding season, common quail showed a high OTUs richness (147 OTUs), although it has to be taken into account that sampled birds were not a flock per se (i.e., they include birds captured in different dates and probably that foraged in the different places), so these data are not comparable with those of other species.

**Figure 4:**
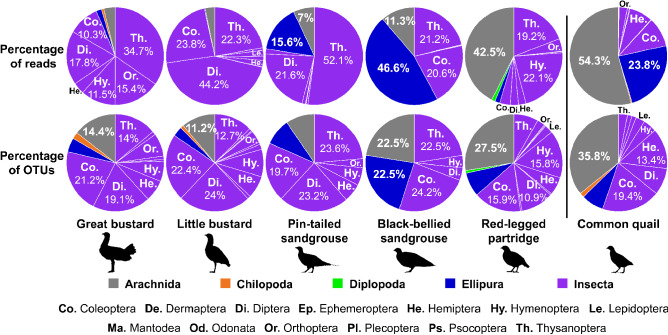
Mean percentage of reads (a measure of abundance) and OTUs (a measure of richness) of each phylum of Arthropoda present on the diet of studied farmland bird species, with phylum Insecta data broken down into orders. Percentages > 10% are detailed numerically. Among Insect’s orders, those with a percentage > 2% are identified. Note that samples of great bustard, little bustard, pin-tailed sandgrouse, black-bellied sandgrouse, and red-legged partridges were collected during the non-breeding season, whereas samples of common quail were collected during the breeding season. The average number of OTUs per species and diet taxa is provided in Supplementary Table S3 online.

Regarding the arthropods that compose the diet of these species, there were large differences between species (Fig. 4). The order Thysanoptera (Insecta) was represented in the diet of all species sampled in the non-breeding season (Fig. 4), although considering its contribution to the total diet, it represented only 5.6% of total reads in little bustard, 3.1% of total reads in great bustard, and less than 0.85% of total reads in the other species. In the case of the bustard species, insects were the most common prey, especially those of the orders Coleoptera, Diptera, and Thysanoptera, and for Great bustard also Hymenoptera and Orthoptera (Fig. 4). Sandgrouse rarely fed on arthropods and the small number of arthropods in their diet was mainly composed of thrips (Thysanoptera) in the case of the pin-tailed sandgrouse, and of springtails (Ellipura) in the case of the black-bellied sandgrouse. Red-legged partridges also mainly fed on plants, although when they fed on arthropods, they mostly took arachnids, hymenopters, and thrips. During the breeding season, common quail showed a very different diet (Fig. 4), with a large proportion of its diet composed of organisms of taxa Arachnida and Ellipura, while insects represented less than 25% of the reads. In case of red-legged partridges and common quail, the species with higher intake of arachnids, the obtained arachnid reads were mainly of spiders (Araneae order (87% and 92% for partridges and quails, respectively). In other species the percentage of spider reads among arachnid reads was lower (great bustard = 74%; pin-tailed sandgrouse = 51%) or extremely low (little bustard = 5%; black-bellied sandgrouse = 0%). Unfortunately, it was not possible to identify arachnid readings that did not belong to spiders. We also found some taxa in the studied species diet that are considered as toxic. These toxic species included *Hymenoptera* (mostly ants) in the diet of all species, *Scolopendra* in the diet of great bustard (in 2 of 6 flocks) and little bustard (in 3 of 14 flocks), another *Chilopoda* in the common quail’s diet and an organism of *Meloidae* family in little bustard's diet (in 1 of 14 flocks).

**Figure 5:**
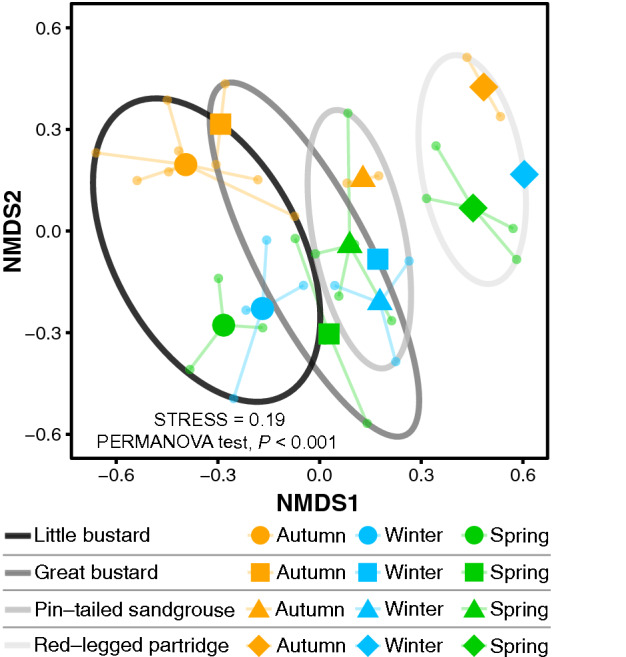
Graphical illustration of the NMDS base on diet OTUs of little bustard, great bustard, pin-tailed sandgrouse, and red-legged partridge represented by season. Large symbols represent the centroid of the flocks of each species in each season, while small dots represent data of each different flock per species and season. The circles group data of each species.

The NMDS and PERMANOVA analyses of seasonal diet differences for great bustard, little bustard, pin-tailed sandgrouse, and red-legged partridge showed significant differences between autumn, winter, and spring (*F*_11,36_ = 2.08, *P* < 0.001; Fig. 5). Those differences were also found for little bustard (*F*_2,13_ = 2.08, *P* < 0.01) and pin-tailed sandgrouse (*F*_2,9_ = 1.61, *P* < 0.01) when species were analysed separately by PERMANOVA testing. In the case of great bustard (*F*_2,5_ = 0.45, *P* = 0.13) and red-legged partridge (*F*_2,6_ = 1.27, *P* = 0.13) we did not find significant seasonal differences, although this could be due to the small sample size (6 flock of great bustard and 7 flock of red-legged partridge, with a single flock of each species sampled in winter). In all species, the autumn diet was the most different (Fig. 5).

**Figure 6:**
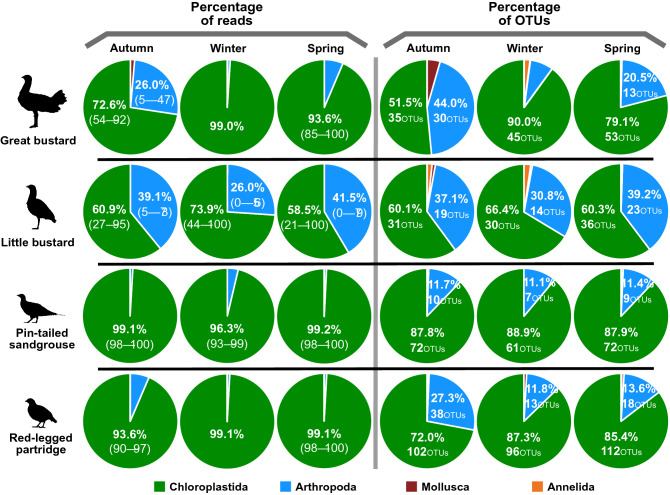
Mean percentage of reads (a measure of abundance) and OTUs (a measure of richness) of the Chloroplastida, Arthropoda, Mollusca, and Annelida taxa present on the diet of great bustard, little bustard, pin-tailed sandgrouse, and red-legged partridge in autumn, winter and spring. Percentages > 10% are detailed numerically and in the case of reads, SD is indicated in brackets. In the OTUs graphs, OTUs richness is also indicated. For great Bustard and red-legged Partridge, only one flock was sampled in winter so no SDs are provided.

Although the OTUs composition on little bustard and pin-tailed sandgrouse varied significantly between seasons (Fig. 5), the proportions of plants and invertebrates in their diet did not change as much. The proportion of plants and arthropods in the little bustard´s diet was similar in autumn and spring, with the proportion of arthropods (percentage of reads) being lower in winter. Pin-tailed sandgrouse fed slightly more on arthropods in winter than in the other two seasons (percentage of reads; Fig. 6) although, in general, arthropods did not fare much in their diet. Red-legged partridges also rarely fed on arthropods during the non-breeding season, although these were more common in their autumn diet. There were clear differences in the proportion of plants and invertebrates (percentage of reads) between seasons in great bustard diet, with higher consumption of arthropods in autumn, while in winter they barely fed on plants (according to the percentage of reads; Fig. 6). In all species, the total OTU richness was very similar in the three seasons (although it was a bit lower in winter; Fig. 6). In this respect, the great bustard was also the species that showed the largest differences.

**Figure 7:**
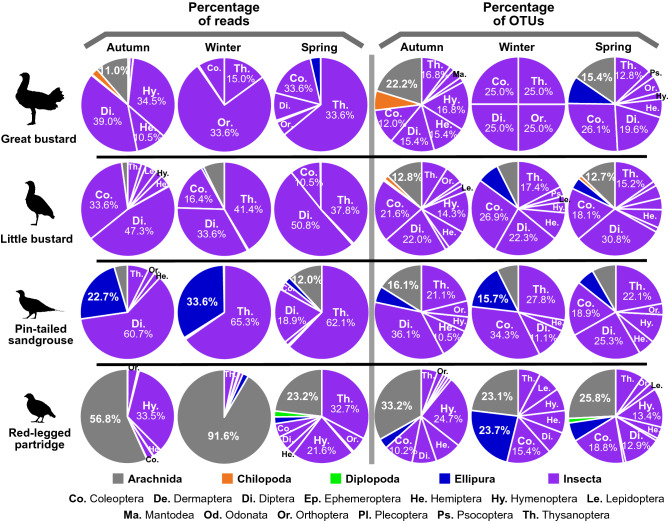
Mean percentage of reads (a measure of abundance) and OTUs (a measure of richness) of each phylum of Arthropoda present on the diet of great bustard, little bustard, pin-tailed sandgrouse, and red-legged partridge in autumn, winter, and spring, with phylum Insecta data broken down into orders. Percentages > 10% are detailed numerically. Among Insect’s orders, those with a percentage > 2% are identified. For great bustard and red-legged partridge, only one flock was sampled in winter so no SDs are provided.

Unlike the plant/invertebrate proportions in the diet, there were strong differences in the proportion of consumed arthropod taxa between seasons (percentage of reads) in all species, although the consumed arthropod taxa richness (percentage of OTUs) did not change as much (Fig. 7). These differences were largely due to the fact that in all species the importance of thrips (Thysanoptera) was quite low in autumn while they contributed greatly to arthropod diets of pin-tailed sandgrouse and little bustard during winter and spring and of great bustard and red-legged partridge during spring (percentage of reads; Fig. 7). Great bustard showed the largest seasonal differences in arthropod consumption, with high importance of Diptera and Hymenoptera in autumn and very high importance of Orthoptera in winter (although all reads belong to a single OTU). Regarding the little bustard´s diet, Coleoptera and Diptera were important in all seasons. In pin-tailed sandgrouse diet, springtails (Ellipura) were important in autumn and winter, Diptera was important in autumn and spring diet, and arachnids appeared also with 12% of arthropod reads in the spring diet (Fig. 7). Red-legged partridge’s arthropod diet was largely composed of arachnids (mostly spiders) in autumn and winter. In autumn, Hymenoptera also made up a large part of its arthropod diet.

## Discussion

Foraging strategies and diet selection play an essential role in individual survival and reproduction^28^, and their study is particularly relevant when it comes to endangered species such as steppe birds or declining gamebirds of high socioeconomic importance. In this work, we used, for the first time, a metabarcoding approach to study the diet of these species, which has provided new and relevant data on their diet composition, including the first detailed description at order level of the arthropod diet of pin-tailed sandgrouse, black-bellied sandgrouse, red-legged partridge, and common quail. Our results showed clear differences between studied species’ diets, with the highest diet similarities among the species most closely related phylogenetically. Little bustards´ diet was more similar to that of great bustards (both species belonging to the *Otidae* family), and contained a large proportion of arthropods (Coleoptera, Diptera, and Thysanoptera and for great bustard also Hymenoptera and Orthoptera) compared with other studied species. Pin-tailed sandgrouse diet was more similar to that of black-bellied sandgrouse (both species belonging to the *Pteroclidae* family), being composed mostly by plants, with small proportions of arthropods (Thysanoptera, Ellipura, Diptera, Coleoptera, and Arachnida) in both species. Red-legged partridge diet was also mostly composed of plants and a small proportion of arthropods (Aracneae, Thysanoptera, and Hymenoptera), although results showed high arthropod OTU richness. In the case of common quail, arthropods were an important part of its diet during the breeding season, especially arachnids (mostly spiders) and springtails (Ellipura). Our results also provided reliable data on differences in diet richness between species, which was very constant among intraspecific flocks, suggesting that diet may be a characteristic of each species. The little bustard was the species with the lowest non-breeding diet richness, showing half of pin-tailed sandgrouse plant OTUs, even if usually both species constitute mixed flocks during the non-breeding period^29^. This result probably indicates a high specificity regarding each species diet, and thus relatively low trophic plasticity. On the other hand, the red-legged partridge showed by far the highest diet richness, even when data was broken down into seasons, which probably indicates much greater flexibility in its food selection. This species can exploit many more habitats than the other species, such as vineyards, scrublands, and olive groves^20^.

Regarding the arthropods that compose the diet of these species, we found some taxa that had not been described (or only occasionally) previously in these species' diets^23,24,30^, such as thrips (Thysanoptera), arachnids (Arachnida) or springtails (Ellipura), and for which our results indicate that they are an important component of their diet. These organisms are often completely digested and therefore underestimated when the diet is analyzed through visual identification in fecal samples^25^. The intake of smaller arachnids (e.g. mites) and springtails (Ellipura) is however likely accidental, and associated with an intake of other trophic resources where these small organisms were present. We found large proportions of thrips within the arthropods that compose the diet of little bustard, great bustard, pin-tailed sandgrouse, black-bellied sandgrouse, and red-legged partridge. Thrips were present in all the samples analyzed in this study and were particularly frequent during winter and early spring. Thysanoptera is an order of very small insects, many of them phytophagous, which develop their life cycle in plants^31,32^. Considering their small size (0.3–14 mm) and cosmopolitan distribution^32^, birds may have also consumed them indirectly through the accidental intake of plants colonized by thrips. In the case of bustards, the species with the highest intake of thrips, this could be due to the high intake of legumes that these species have in winter^24,30^. However, a positive selection of plants colonized by thrips because they contain a higher level of nutrients than plants without thrips cannot be ruled out. In the case of both sandgrouse diet, thrips represented a lower proportion of their arthropod diet, possibly because sandgrouse feed mainly on seeds rather than on leaves^20,22^. This could also explain why thrips were less frequent in all species in their autumn diet, when crop fields are mostly bare or newly sown. Future studies should specifically assess whether these bird species actively select plants colonized by thrips. If this was the case, steppe birds could be providing an important ecosystem service in terms of pest control^33^, since many thrips species present invasive characteristics and could be vectors of different viruses that are detrimental to crops^31,32^.

Our study also revealed the high importance of spiders in the common quail's diet, which was as yet unknown; our results clearly showed a large intake of this invertebrate phylum by this species. Birds have been described as important predators of spiders in forest habitats, but not in open ecosystems^34^. Common quails may feed on spiders because they have high nutritious value and lack defensive chemicals (unlike ants) that might be toxic or repelling^34^.

In that context, we found that the arthropod diet of some species contained, in low proportions, some taxa such as ants, Scolopendra spp. and Meloidae (that include toxic species) that might be consumed for auto-medication, and used as antiparasitic food^35–37^. Previous studies have demonstrated the use of some plant species and insects of the Meloidae family as antiparasitic agents by great bustards^38,39^. However, as far as we know, this is the first time that Meloidae is described in the diet of the little bustard and that Scolopendra spp. is reported in the diet of both great bustard and little bustard. In the case of ants, they were mainly consumed in autumn. During this season, due to the emergence of winged ants, ant availability may be particularly high and these may be taken opportunistically as a readily available food resource, but ants could also be consumed for antiparasitic use^36^.

Our results also showed that diet composition varied across seasons, with important changes in the proportion of consumed arthropod taxa, being less diverse in winter than in autumn and spring. These results probably reflected differences in arthropod availability in the different seasons^40^. However, except for the great bustard diet, the ratio between plants and invertebrates in the diet did not change as much, and plant and arthropod richness was quite constant throughout the non-breeding season. The results obtained for the great bustard (a strong reduction in the proportion of arthropods in its winter diet) are in line with previous knowledge^41^, showing that the winter diet of great bustards was based almost exclusively on vegetable matter. On the other hand, the low variation in arthropod taxa richness in the little bustard diet between the seasons may reflect this bird's high affinity for certain prey and low trophic plasticity. The red-legged partridge, by contrast, showed both high plant taxa richness and high arthropod taxa richness, although the proportion of arthropod reads in its diet was low. This showed the low trophic specificity and high trophic plasticity of this species, at least in comparison with the other studied species. There were probably also important changes in the proportions of consumed plant taxa, but unfortunately, our methodology did not allow us to break down plant data^42^, and this should be assessed in future studies.

Our results have relevant implications for the little bustard, a threatened species in deep decline^43,44^ and the species with the least diverse diet (especially regarding plants) and with the highest proportion of arthropods on his diet during the non-breeding season. According to our results, the little bustard diet during the non-breeding season may include a higher proportion of arthropods than estimated by previous studies based on visual analysis of feces^30^. It should be noted that most of the arthropods described in their winter diet (thrips, flies, and arachnids) belong to taxa with few chitinous structures, potentially less detectable by a visual study of remains in the feces. By contrast, the proportion of Coleoptera in their winter diet was small. With all this in mind, the little bustard is possibly the most vulnerable species to changes in trophic resources, such as the loss of weed and arthropod biodiversity and abundance resulting from agriculture intensification^15^. During winter, this species has been seen using legume fields, stubbles and fallow lands^45–47^. Alfalfa fields provide an important plant trophic resource that the species uses throughout the year^30,45,46^ and also provide a high abundance of arthropods^46^. Stubbles, although they maintain a lower abundance of arthropods, are positively selected because their vegetation structure facilitates the location and capture of prey^46^. Moreover, natural and semi-natural environments (such as vegetation growing in fallow land) play an essential role for arthropod communities, providing over-wintering areas for that group and maintaining an overall greater abundance and richness of arthropods than crop lands^48,49^. A strong association between the decline in little bustard abundance and the decline in the fallow surface has been shown^8^, suggesting a potential link between these two factors. This relationship between arthropods and fallow land reinforces the need for a new CAP that guarantees the maintenance of fallow land in the European agroecosystems if little bustard populations, as well as those of other farmland birds, are to be conserved^8,13^.

The proportion of arthropods in the non-breeding diet of sandgrouse species was very small, consistent with a high dependence on vegetable trophic resources. Pin-tailed sandgrouse and black-bellied sandgrouse are mainly granivorous species^20^, so they rely on the availability of seeds and seed banks. Seed availability is highly variable between habitats and among seasons, being also strongly influenced by agricultural intensification^18,50^. Within arable land, natural and semi-natural vegetation growing in grassy field boundaries and fallow land provides an important stock of seeds, being thus important habitat elements for these species^50^.

Within these landscapes in continuous intensification, updated knowledge on species diet becomes essential to address their conservation. In this line, our results provided relevant data on the diet of these declining species throughout the non-breeding season (the least studied season so far), especially with regard to invertebrates. Moreover, they showed the importance of arthropods in these species' diets, proving the need to adequately maintain the arthropod populations within agricultural landscapes. This could be crucial to address the conservation of endangered species. Unfortunately, we were not able to obtain a balanced number of samples for all species, and in the case of the black-bellied sandgrouse (2 flocks) and common quail (1 group), the generalization of our results should be taken with caution. In addition, due to the bias that may occur throughout the process of extraction, amplification and sequencing of fecal DNA, the percentages of dietary composition presented in this study should be taken as indicative and in no case as absolute, especially the plant / arthropod ratio. In the future, this study should be complemented by a more detailed assessment of the plant diet, which would help to clarify the extent to which these birds feed on cultivated crops, including pesticide coated seeds^10^, and the role that fallows and semi-natural habitats play as food sources. Besides, the study of diet throughout the breeding season should be also assessed, as it is known that in the breeding season diet could change significantly^23,41^. Moreover, chicks might need a diet richer in protein for growth, as highlighted for great bustard, little bustard, and red-legged partridge, whose chicks rely on arthropod diets^23,24,51^, but the trophic requirements of sandgrouses´ chicks have not yet been described in depth. To properly address the conservation needs of these species, especially of threatened steppe bird species, agriculture should be managed in a way that ensures fulfilling trophic needs. This could be achieved in a sustainable way by promoting agri-environmental schemes attractive and profitable for farmers. These measures should be focused on increasing the complexity of the ecosystems, to shift these ecosystems towards a mosaic landscape, which can provide a wide variety of arthropods, plants, and seeds throughout the year.

## Methods

### Ethics statement

All actions were performed according to Spanish law. In this study we did not use experimental animals and for all analyses non-invasive samples (feces) were used. In addition, fecal samples of all species, except for common quail fecal samples, were collected in a manner that did not disrupt the birds' routine (see below). Common quail fecal samples were collected during ringing seasons. Common quails were caught by qualified persons (expert ringers endorsed by the Aranzadi Ringing Scheme) and with the relevant administrative permission, issued by the Provincial Council of Alava (permission number: 17/009), and all of them were released in perfect condition after ringing them. None were retained for the purpose of collecting their feces; we only collected fecal samples from those common quails that defecated during the ringing process.

### Sampled species

The study was carried out in Spain, a region where agricultural pseudo-steppes are home to the most important European populations of some threatened steppe-bird species, such as little bustard, great bustard and pin-tailed sandgrouse, and which also hold a big population of black-bellied sandgrouse^21,43^. Most of these species are classified as vulnerable in Spain (Real Decreto 139/2011). Moreover, all of them have suffered a population decline to some extent throughout the last decade^27,43,44,52^, with strong declines of −48% (2005–2016) and −43% (2005 and 2019) in the case of little bustard and black-bellied sandgrouse^27,43,44^. The Iberian Peninsula also holds most of the wild (native) red-legged partridge population^53^ and an important common quail population^54^. Red-legged partridge and common quail are game species of high socio-economic importance, especially the former, which is the main small game-bird species in Spain^55^. All four steppe-bird and the two game-bird species rely on the pseudo-steppes and extensive farmland ecosystem and its resources^21^ but, despite their ecological and socio-economic importance, their trophic ecology is poorly studied and it has never been assessed using a molecular approach^20,22–24,30,41,56^.

### Sample collection

**Figure 8:**
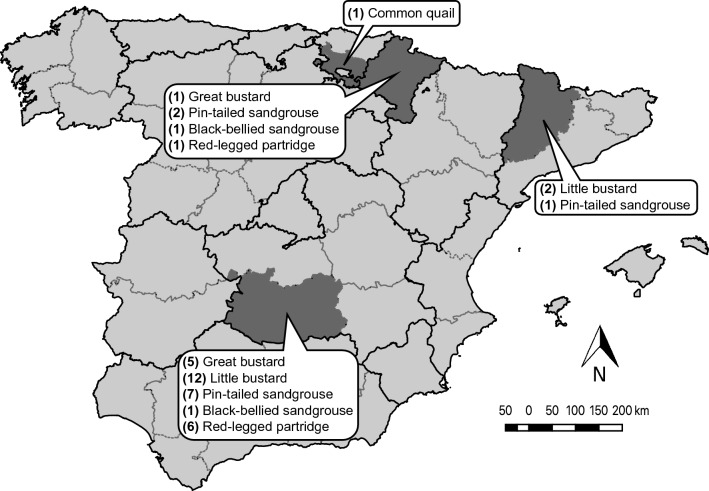
Map of Spanish regions with the spatial distribution of each species samples. The number of flocks per species sampled in each region is shown in brackets. This map was generated using QGIS v3.10^57^.

All samples were collected in Spain, most of them in the province of Ciudad Real (39° 0′ N, 4° 0′ W), but also in Navarra (42° 49′ N, 1° 39′ W), Lleida (41° 48′ N, 1° 28′ W), and Araba (42° 50′ N, 2° 45′ W) (the latter only for common quail; Fig. 8). Most samples were collected during the non-breeding season (autumn, winter, and early spring) of 2017 and 2018, except for the little bustard samples from Lleida (collected in winter and early spring of 2011, 2012, and 2013) and common quail samples (collected throughout the breeding season -late spring and summer- of 2017). The breeding season of these species extends from April to August^20^, so here we use the term breeding season to cover this period and the term non-breeding season to cover the period from September to early April. We collected feces samples in roosting places used to spend the night, so as not to alter the birds' routine, except in the case of quails, whose feces were collected during the scientific ringing process of these birds (work done by expert ringers from the Aranzadi Science Society, with the corresponding administrative authorization). During the non-breeding period, studied species (except for quails) usually gather in flocks. We sampled in roosting places early morning where we had located flocks at dusk. Within roosts, we sampled feces that were at least 2 m apart to avoid sampling the same individuals twice. For quail, to be consistent, we group all individual birds ringed within a single breeding season within a single “group”, although this species does not form flocks during the breeding season and the samples are not even from the same date. This should be considered when our results will be interpreted. We collected fecal samples of a total of 40 flocks, distributed as follows: six flocks of great bustard, 14 flocks of little bustard, 10 flocks of pin-tailed sandgrouse, two flocks of black-bellied sandgrouse, seven flocks of red-legged partridge, and a group of common quail. In the case of the two little bustard’s flocks from Lleida, they were not flocks *perse*, as they include samples from different years. When possible, feces of 20 individuals per flock were collected. In the field, samples were collected in individual zipper bags, avoiding cross-contamination, and stored in cold conditions until they reached the laboratory, where they were frozen at −80 ºC for a week and then kept at −20 ºC until they were processed.

### Sample processing

DNA extractions were performed using the QIAamp DNA Stool Mini Kit from QIAGEN (Ref. 51504). From 1 to 20 (14 on average) DNA samples were obtained per flock (Supplementary Table S1 online). We used a NanoVue Plus spectrophotometer (GE Healthcare) to measure the concentration (μg/ml) and quality (A260/280 and A260/230 ratios) of each DNA sample. Although we extracted DNA from each fecal sample, we considered each flock as a unique sample for analyses, and during the preparation of the libraries, samples of the same flock were mixed by adding them the same index. First, samples from each flock were mixed according to DNA quantity and quality in three pools of 1–7 samples, one of high, one of medium, and one of low quantity and quality (Supplementary Fig. S2 online). Subsequently, PCR amplification of each sample pool of each bird flock was performed using the Eukaryotic mini-barcode miniB18S_81 designed by Cabodevilla et al. (2020)^42^ at the Analytical Services (SGIker) of the University of the Basque Country (UPV/EHU). We included PCR blanks within the library constructions and did not detect any contamination. Samples were purified and a second reaction was performed to index each amplified product and attach Illumina adaptors using the Illumina Nextera v2 kit. We added the same index to the three pools of each bird flock, using a different index per flock (Supplementary Fig. S2 online). The amplification was carried out according to Cabodevilla et al. (2020)^42^, using the same DNA quantity, reaction mix, and thermocycler conditions. PCR outputs were sequenced in an Illumina MiSeq NGS platform (sequencing of 2 × 150 bp paired-end reads) with the MiSeq Reagent Kit v2, following the manufacturer's instructions.

### Bioinformatics and statistical analyses

The sequencing output was processed using the Cutadapt^58^ and Usearch^59^ software packages. First, the adapters were cut and forward and reverse sequences were merged (77.34% of sequences merged correctly). The sequences were then cleaned, filtering them by expected errors (*fastq_filter* function of Usearch, allowing a single expected error; 97% passed), the unique sequences and the OTUs were identify using *fastx_uniques* and *cluster_otus* functions of Usearch respectively, and the OTU table was built based on 97% identity using the *usearch_global* function. In other words, amplicons with 3% similarity were grouped into the same OTU. In addition, we deleted all OTUs with less than 3 sequences to deal with singletons. We did not use any specific analysis to eliminate chimeric sequences. Subsequently, we used the *sintax* function of Usearch and the SILVA 18S v123 reference dataset^60^ to predict the taxonomy of the obtained OTUs. We used a bootstrap cutoff of 0.8 to identify taxonomy at the kingdom, phylum, and class levels and a bootstrap cutoff of 0.7 to identify taxonomy at the order level. We did not include sequences of the 16S gene on our reference data set, since used primers do not amplify prokaryotic organisms^42^. Then we filtered the OTU table, selecting only those OTUs that could belong to the studied birds’ diet, OTUs belonged to Chloroplastida, Arthropoda, Mollusca (only those of Gastropoda class), and Annelida (only those of Clitellata class) taxa. Among OTUs belonging to the phylum Arthropoda, those OTUs belonging to the classes Maxillopoda and Ostracoda (organisms inhabiting the water) and to the order Phthiraptera (ectoparasites) were also omitted, as these were probably not part of the steppe birds’ diet but they are environmental contamination. Once filtered, we normalized the OTU table transforming the data within each flock to percentages, using the Total Sum Normalization method (TSS)^61^: we estimated the percentage that the reads of each OTU represents among the total reads obtained for each flock (reads of specific OTU/total read of this flock × 100). This makes data comparable among flocks and species.

We used the OTU table to describe the diets of the species analyzed based on the mean values and SD of the percentage of reads and the percentage of OTUs. The percentage of reads represents a measure of the abundance of the OTUs of specific taxa while the percentage of OTUs represents a measure of richness within a specific taxon. Various studies have indicated that the number of reads might be used as a proxy of relative taxa abundance^62–64^, but many others have shown that this is not always the case. Mock samples can be used to validate the semi-quantitative capability of these primers, but were not used in this study, so quantitative aspects should be considered with caution. Although reads abundance is not commonly used in zoology and ecology, it is quite common in other disciplines such as mycology or microbiology, in which many important studies use this approximation^65–68^. OTUs should not be considered as species, as we do not know the resolution within each taxon. Here, OTUs represent an index of richness, which we find valuable and comparable across flocks and species. For common quail it was not possible to obtain mean values and SD since, as we grouped all individual samples in a single group, we only obtained one data. We used these values to describe the composition of plants (sequences belonging to the Chloroplastida kingdom) and invertebrates (Annelida, Arthropoda, and Mollusca phyla, from the Metazoo kingdom) of the diet of the studied bird species, describing in detail the composition of the animal part of the diet (Arthropoda phylum classes belonging to the diet and Insecta class orders). In the case of the plants, the used mini-barcode shows very low resolution capacities at the order level, so we decided to include such results at the kingdom level since most OTUs belonged to the same class (Embryophyta) and same phylum (Phragmoplastophyta)^42^. As common quail samples were collected at a different time of year than those for other species, the results for this species were considered separately. We carried out an ANOVA in R v3.6.1^69^ to check if there were differences in OTUs richness between bird species. Our response variable was the number of OTUs per bird flock and the explanatory variable was the species. We break down the results of the ANOVA by species applying a Tukey post-hoc test, using the *lsmeans* package in R^69,70^. In addition, in the case of great bustard, little bustard, pin-tailed sandgrouse, and red-legged partridges, we also made a comparison of diet by season, comparing their diet in autumn, winter, and spring. We classified samples in relation to season within the non-breeding season as follows: from September to November were considered as autumn, from December to February as winter, and from March to April as spring. In addition to the direct comparisons, differences in diet between species and between seasons within species were tested by Non-metric multidimensional scaling (NMDS) and Permutational multivariate analysis of variance (PERMANOVA) models (*metaMDS* and *adonis* functions of *vegan* v2.4-2 package in R)^69,71^. For this purpose, the database was transformed by applying a fourth root. NMDS is an ordination technique that uses a Bray–Curtis matrix of ranked similarities and displays samples in low-dimensional space while retaining as nearly as possible the similarity rankings between samples.

## Supplementary Information

Supplementary Information.

## Data Availability

The datasets analysed during the current study are available from the corresponding author on reasonable request.
