# Supplementary material for: Metabarcoding insights into the diet and trophic diversity of six declining farmland birds
Source: Sci Rep. 2021 Oct 26;11:21131. doi: 10.1038/s41598-021-00519-9 (PMC8548310; doi:10.1038/s41598-021-00519-9)
Supplement: Supplementary file 1 — Supplementary Information. [file 41598_2021_519_MOESM1_ESM.pdf]

Supplementary Information

Metabarcoding insights into the diet and trophic diversity of six declining farmland birds.

Xabier Cabodevilla[1,2,*], François Mougeot[2], Gerard Bota[3], Santi Mañosa[4], Francesc Cuscó[4], Julen Martínez-García[1], Beatriz Arroyo[2†], María J. Madeira[1†]

[1] Department of Zoology and Animal Cell Biology, Faculty of Pharmacy, University of the Basque Country (UPV/EHU), Paseo de la Universidad 7, 01006 Vitoria-Gasteiz, Alava, Spain

[2] Instituto de Investigación en Recursos Cinegéticos (IREC) (CSIC-UCLM-JCCM). Ronda de Toledo 12, 13005 Ciudad Real, Spain

[3] Landscape Dynamics and Biodiversity programme, Forest Science and Technology Center of Catalonia (CTFC), Solsona, Catalonia, Spain

[4] Departament de Biologia Evolutiva, Ecologia i Ciències Ambientals. Institut de Recerca de la Biodiversitat (IRBio), Universitat de Barcelona, Avinguda Diagonal 643, 08028 Barcelona, Catalonia, Spain.

***Correspondence to:** xabier.cabodevilla@ehu.eus

[†] **Shared last authorship**

Table S1. DNA samples collected by species flock, with information on flock's origin and sampling season.

Species	Flock	DNA	Province	Season
**Little bustard**	1	10	Ciudad Real	Early spring
**Little bustard**	2	17	Lleida	Winter
**Little bustard**	3	16	Lleida	Early spring
**Little bustard**	4	16	Ciudad Real	Winter
**Little bustard**	5	15	Ciudad Real	Winter
**Little bustard**	6	19	Ciudad Real	Winter
**Little bustard**	7	11	Ciudad Real	Autumn
**Little bustard**	8	20	Ciudad Real	Autumn
**Little bustard**	9	14	Ciudad Real	Autumn
**Little bustard**	10	13	Ciudad Real	Autumn
**Little bustard**	11	19	Ciudad Real	Autumn
**Little bustard**	12	18	Ciudad Real	Early spring
**Little bustard**	13	20	Ciudad Real	Autumn
**Little bustard**	14	18	Ciudad Real	Autumn
**Great bustard**	1	6	Ciudad Real	Early spring
**Great bustard**	2	7	Navarra	Early spring
**Great bustard**	3	17	Ciudad Real	Winter
**Great bustard**	4	17	Ciudad Real	Autumn
**Great bustard**	5	11	Ciudad Real	Autumn
**Great bustard**	6	1	Ciudad Real	Early spring
**Pin-tailed sandgrouse**	1	11	Navarra	Early spring
**Pin-tailed sandgrouse**	2	16	Navarra	Early spring
**Pin-tailed sandgrouse**	3	15	Ciudad Real	Early spring
**Pin-tailed sandgrouse**	4	13	Lleida	Winter
**Pin-tailed sandgrouse**	5	16	Ciudad Real	Winter
**Pin-tailed sandgrouse**	6	17	Ciudad Real	Autumn
**Pin-tailed sandgrouse**	7	16	Ciudad Real	Winter
**Pin-tailed sandgrouse**	8	14	Ciudad Real	Autumn
**Pin-tailed sandgrouse**	9	15	Ciudad Real	Early spring
**Pin-tailed sandgrouse**	10	15	Ciudad Real	Early spring
**Black-bellied sandgrouse**	1	20	Navarra	Autumn
**Black-bellied sandgrouse**	2	18	Ciudad Real	Winter
**Red-legged partridge**	1	11	Ciudad Real	Autumn
**Red-legged partridge**	2	15	Ciudad Real	Autumn
**Red-legged partridge**	3	14	Ciudad Real	Winter
**Red-legged partridge**	4	11	Ciudad Real	Early spring
**Red-legged partridge**	5	9	Ciudad Real	Early spring
**Red-legged partridge**	6	6	Navarra	Early spring
**Red-legged partridge**	7	9	Ciudad Real	Early spring
**Common quail**	1	16	Alava	Spring-

Table S2. Statistical results of Tukey post-hoc analysis for the ANOVA model for differences on OTUs richness among species.

Species	*Degrees of freedom*	*t.ratio*	*P*
**LB * GB**	34	-1.78	0.40
**LB * PTS**	34	-4.34	**< 0.01**
**LB * BBS**	34	-2.90	**< 0.05**
**LB * RLP**	34	-11.70	**< 0.001**
**GB * PTS**	34	-1.80	0.39
**GB * BBS**	34	-1.62	0.49
**GB * RLP**	34	-8.17	**< 0.001**
**PTS * BBS**	34	-0.51	0.99
**PTS * RLP**	34	-7.34	**< 0.001**
**BBS * RLP**	34	-4.02	**< 0.01**

Table S3. Average number of OTUs per species. Complementary information to Fig. 4.

**Taxonomic level**			**Average number of OTUs per species**					
**Kingdom**	**Phylum**	**Order**	**Great bustard**	**Little bustard**	**Pin-tailed sandgrouse**	**Black-bellied sandgrouse**	**Red-legged partridge**	**Common quail**
Chloroplastida			45.5	31.7	68.8	74.0	107.0	78.0
Mollusca			1.2	0.4	0.3	0.5	0.9	1.0
Annelida			0.2	0.7	0.1	0.5	0.3	1.0
Arthropoda			17.2	18.4	8.5	8.5	22.9	67.0
Arthropoda	Arachnida		3.2	2.1	0.9	2.0	6.1	24.0
	Chilopoda		0.7	0.2	0.0	0.0	0.0	1.0
	Diplopoda		0.0	0.0	0.0	0.0	0.1	0.0
	Ellipura		0.5	0.6	0.7	2.0	1.4	5.0
	Insecta		12.8	15.4	6.9	4.5	15.1	37.0
	Insecta	Coleoptera	3.0	4.1	1.6	1.5	3.6	13.0
		Dermaptera	0.0	0.0	0.0	0.0	0.1	0.0
		Diptera	3.0	4.4	2.1	0.5	2.3	6.0
		Ephemeroptera	0.0	0.1	0.0	0.0	0.1	0.0
		Hemiptera	1.7	1.3	0.6	0.0	1.7	9.0
		Hymenoptera	1.8	1.7	0.6	0.5	4.3	3.0
		Lepidoptera	0.2	0.3	0.0	0.0	0.6	2.0
		Mantodea	0.3	0.1	0.0	0.0	0.1	0.0
		Odonata	0.0	0.0	0.0	0.0	0.1	0.0
		Orthoptera	0.7	0.8	0.3	0.0	0.6	1.0
		Plecoptera	0.0	0.1	0.0	0.0	0.0	1.0
		Psocoptera	0.2	0.1	0.0	0.0	0.0	0.0
		Thysanoptera	2.0	2.3	1.7	2.0	1.6	2.0

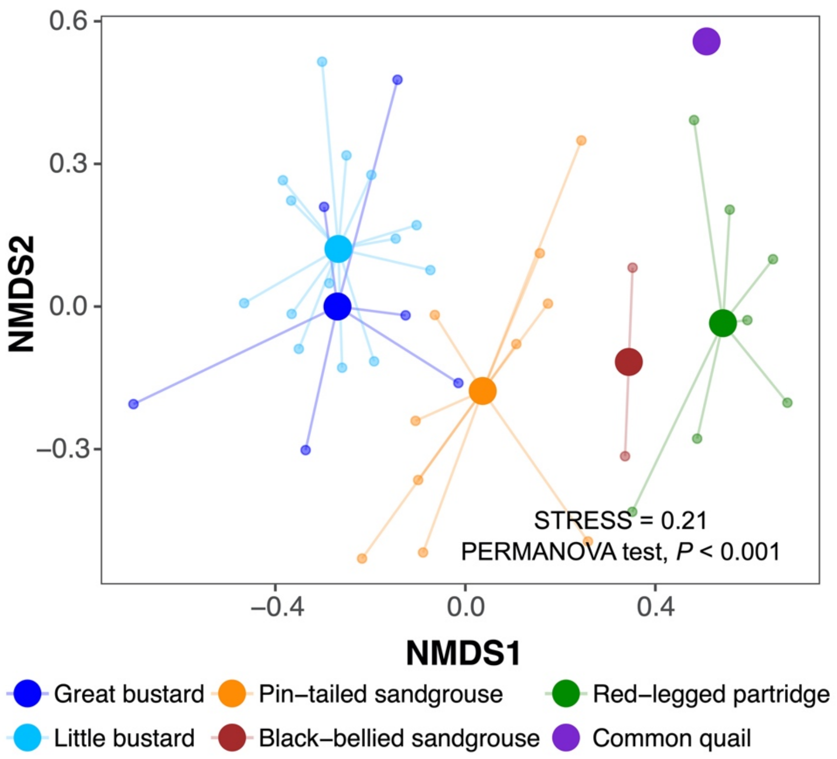

Figure S1. Graphical illustration of the results of the NMDS analysis of the studied birds' diet, based on presence data. Larger dots represent the centroid of the flocks of each species, while smaller dots represent the individual flock data of each species.

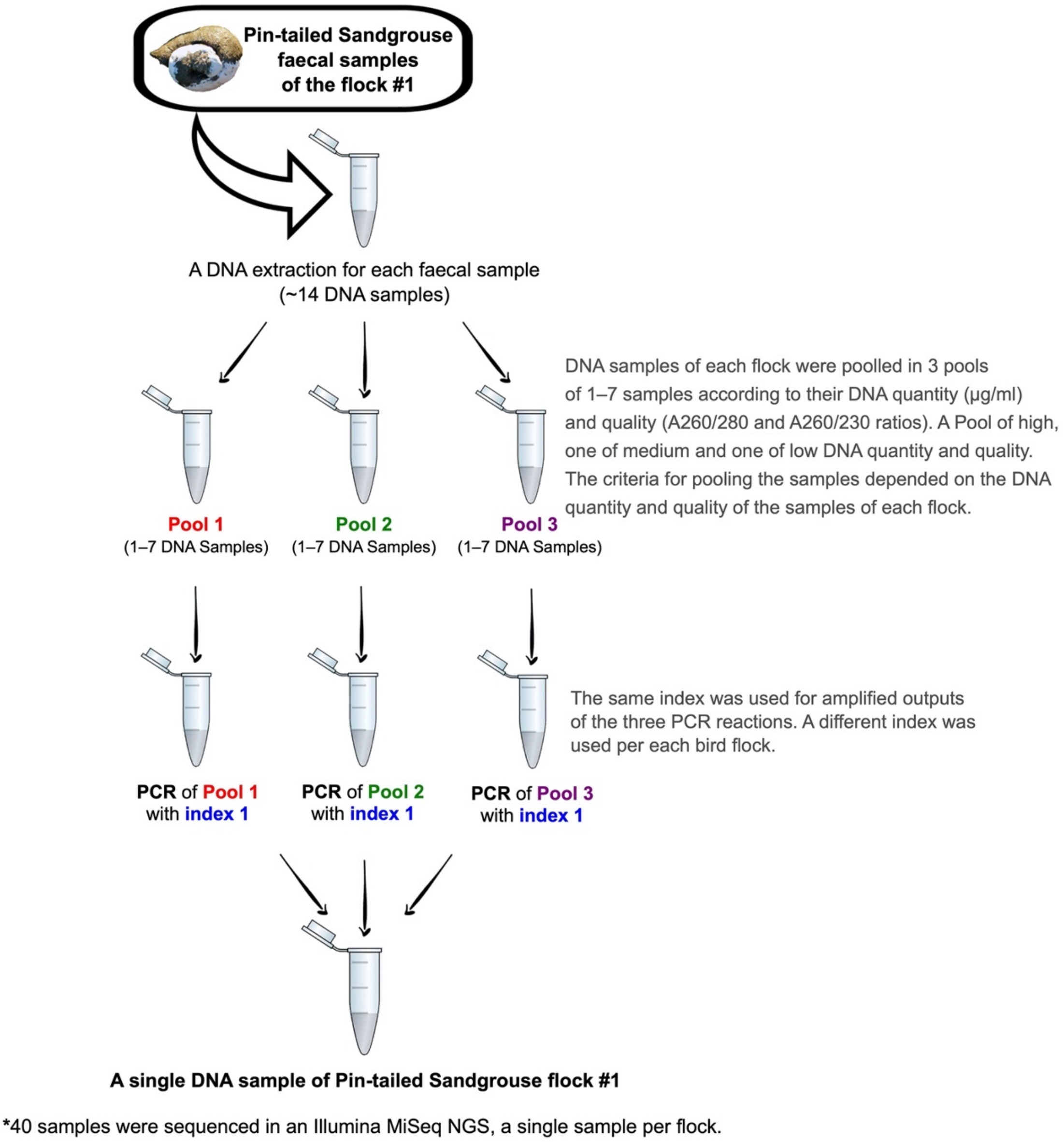

Figure S2. Graphical illustration of library construction from faecal samples.
